# In Situ Atomic‐Scale Observation of Preferential Premelting at Oxide Crystal Defects

**DOI:** 10.1002/smll.74384

**Published:** 2026-07-01

**Authors:** Zaoli Zhang, Zhuo Chen, Yong Huang, David Holec, Yonghui Zheng, Jan Michalička

**Affiliations:** ^1^ Erich Schmid Institute of Materials Science Austrian Academy of Sciences Leoben Austria; ^2^ Department of Materials Science Montanuniversität Leoben Leoben Austria; ^3^ Key Laboratory of Polar Materials and Devices (MOE) Department of Electronics East China Normal University Shanghai China; ^4^ CEITEC Nano Brno University of Technology Brno Czech Republic

**Keywords:** atomic structure, BiFeO_3_, planar defects, DFT, EELS, in situ TEM

## Abstract

Under certain conditions, pulsed laser deposition‐grown BiFeO_3_ thin films can exhibit a high density of non‐stoichiometric line defects and planar defects. The defective regions, characterized by altered lattice periodicity, local atomic configurations, and electronic states, are expected to respond to external stimuli in ways distinct from those of the pristine matrix. Here, we uncover an unexpected phenomenon of atomic‐scale premelting in BiFeO_3_ thin films. Using atomic‐resolution imaging combined with spectroscopic analysis, we reveal that dislocations and stacking faults can act as preferential nucleation sites for premelting. Enabled by fast‐acquisition in situ transmission electron microscopy, we directly visualize site‐specific, atomic‐scale premelting processes in real time. Furthermore, we capture transient atomic configurations associated with evolving local strain. These observations indicate that the coupled effects of local strain accumulation and oxygen‐vacancy enrichment, driven by atomic displacement damage under external stimuli, trigger the formation of an amorphous phase. First‐principles calculations further corroborate the experimental findings. Together, this work reveals a defects‐mediated pathway for amorphization in complex oxides and highlights the critical role of local structural and chemical heterogeneities in governing phase stability under external perturbations.

## Graphical abstract

Under an external stimulus (e.g., an electron beam irradiation), premelting preferentially initiates at the planar defects in crystalline materials, thereby altering the local atomic and electronic structures. [Correction added on July 21, 2026, after first online publication: The graphical abstract figure has been updated.]

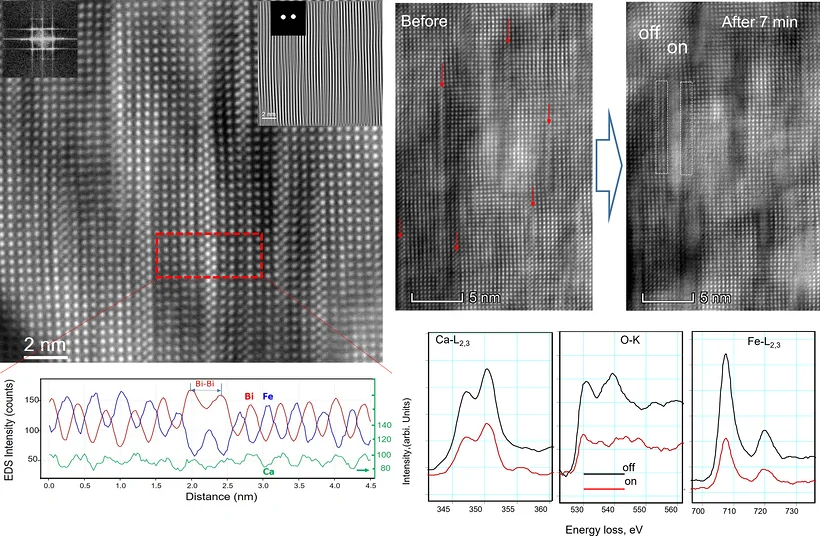

## 1 Introduction

According to classical melting theory [1, 2, 3], melting is a phenomenon that occurs in solid materials, characterized by the localized loss of crystal order, which can drastically alter functionality or degrade performance. It has recently garnered increasing interest in materials research, both theoretically and experimentally [3, 4]. Tracking the origin of melting in crystalline materials is beneficial for controlling the initial processes and tailoring material performance. Many experimental and theoretical efforts have been devoted to understanding the melting of different materials, including in situ investigations of liquid‐to‐solid transitions using high‐resolution transmission electron microscopy (HRTEM) [5, 6] and monitoring the premelting process and the contacting of phase transition, etc. Numerous theories suggest that a similar premelting phenomenon should occur at defects such as grain boundaries, stacking faults (SFs), and dislocations in the bulk [7]. However, the direct experimental evidence is rare, and the detailed atomic‐scale mechanism of premelting remains unclear. This is due to the difficulty of precisely tracking atomic details during melting. Within bulk colloidal crystals, it was reported that premelting occurs at grain boundaries, as well as at dislocations and at the surface [3, 7]. Whether it exhibits similar behavior in crystalline materials is uncertain, given the complex bonds and atomic interactions that may govern the nucleation of solid‐to‐solid and solid‐to‐liquid phase transitions [4, 6]. The origin of pre‐melting at defects (grain boundaries, interstitials, or vacancies) is typically explained by Lindemann and Born's theories [8, 9], which attribute it to vibrational and elastic instabilities in the lattice, thereby triggering melting. However, it is difficult to experimentally visualize or detect vibrational and elastic instabilities in lattices. Further, most previous investigations have yielded little insight into the effects of premelting on the parent microstructures. The loss of local crystal order leads to rearrangements and readjustments of the surrounding atomic configurations, reflected in changes in bond types/length and coordination numbers. Consequently, the corresponding atomic and electronic structures in the parent lattice are also altered. A comprehensive understanding of the underlying atomic mechanism governing reversible transitions beyond classical theory will generate broad, growing interest in fundamental science and applications, as it may provide the necessary insights into the precise control of phase transitions.

With the advent of aberration‐correction techniques and the development of a fast‐recording system, capturing the individual atomic processes and behaviors has become feasible. This has been shown to provide detailed atomic‐scale insights into the dynamic process [10, 11]. Moreover, quantitative atomic‐resolution analysis enabled by the correction technique, i.e., localized atom displacement and strain, may improve our understanding and shed light on the fundamentals of the premelting process in crystalline materials [12].

Bismuth ferrite (BiFeO_3_, thereby abbreviated as ‘BFO’) has garnered considerable attention recently due to its distinctive ferroelectric, magnetic, piezoelectric, and optical properties. It also holds promise for technological applications in information storage and spintronics [13, 14]. One challenge in preventing BFO commercialization is its high leakage current, which is strongly influenced by its structure and localized defects, particularly point defects [15]. Therefore, identifying growth defects and exploring their structural evolution are of practical importance for the material. It is noticed that numerous defects could be incorporated during the synthesis of BFO oxide films, including two‐dimensional defects (i.e., stacking faults) and one‐dimensional defects (i.e., dislocations). Some were previously considered ‘single‐layer’ Bi oxide phases within the unit cell [16]. Nevertheless, they can still be regarded as stacking defects with respect to the integrity of the BFO film. Importantly, these defects have been explored only briefly so far [16, 17, 18], and it remains unclear whether and how they evolve in response to external stimuli. As noted, defects can significantly compromise the structural integrity of BFO films used in devices, thereby degrading film performance. Exploring these defect evolutions is demanding for synthesis and applications.

On the other hand, direct atomic‐scale observation brought by in situ HRTEM [19, 20, 21] enables real‐time tracking of atomic and electronic‐structure evolution and provides important information for tailoring this structure during deposition and for optimizing BFO performance in general. Atomic‐scale in situ HRTEM observations have proven powerful for revealing melting and solidification in Bi oxides [6, 11, 19, 21], and precisely elucidating the atomic‐scale behaviors of these processes under an external electron beam and other stimuli, such as nucleation, crystallization, solid‐liquid transformation, and crystal growth [20]. The valuable information acquired can significantly enhance our understanding of classic nucleation and growth in crystalline materials, as well as solid‐liquid transformation behaviors under external stimuli.

In this study, by coupling atomic‐resolution HRTEM imaging with a fast‐recording system, we observe the structural characteristics of defects in BFO and demonstrate their evolution under external stimuli, thereby further elucidating the fundamental origin of premelting.

## 2 Results

### 2.1 Atomic and Electronic Structures at Defects

**FIGURE 1:**
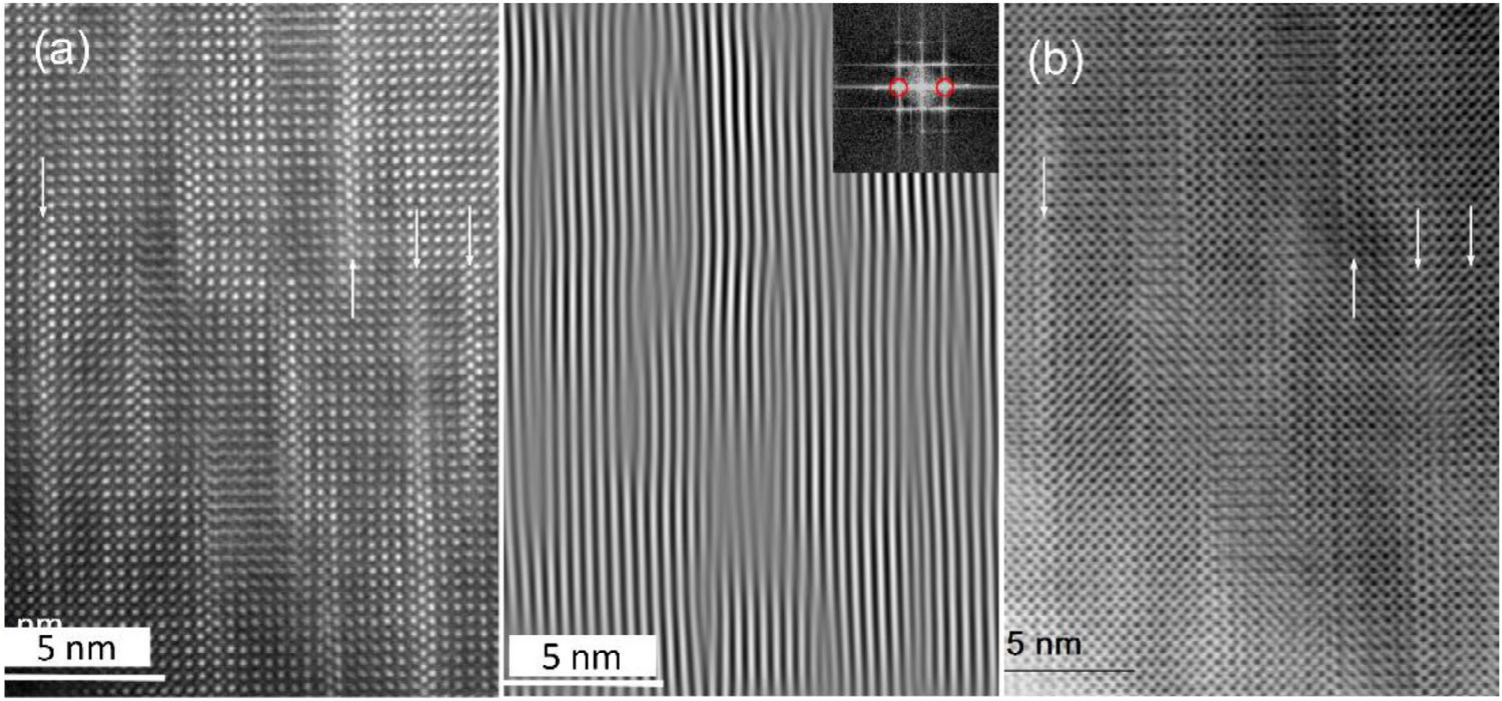
A typical high‐resolution STEM HAADF image (a), and corresponding STEM‐BF image (b). A Bragg‐filtered image using selected reflections in the FFT pattern (inset in (a)) shows the distribution of faulted planes (or ‘planar defect’). The arrows indicate the identical locations in the Z‐contrast (a) and STEM phase‐contrast images (b) of the atom columns.

The microstructure and atomic structure of this Ca‐doped BFO film were comprehensively examined. Figure 1a shows a typical atomic‐resolution scanning transmission electron microscopy (STEM) high‐angle annular dark field (HAADF) image of the as‐deposited films, where cation atom species (Bi, Fe) are imaged unambiguously with the very bright contrast (Bi) and weakly bright contrast (Fe). In contrast, anion atoms (i.e., O) are hardly seen in this mode. Across the entire image, numerous white, ‘stripe’‐like contrasts are distributed in the films when viewed along the [100]_pc_ direction. The magnified atomic structure reveals that the strong, bright contrast also appears at column positions assigned to Fe atoms, forming one‐ or two‐atom‐thick faulted planes throughout the film. This implies that Bi atoms partially replace Fe atom columns at faulted planes. Figure 1b exhibits the corresponding STEM‐ BF (Bright field) image, in which both Bi and Fe contrasts are clearly seen. Such faulted planes (or ‘planar defect’ in general), to which we refer as the ‘stacking fault’ (abbreviated as ‘SF’), can be better visualized in the Bragg‐filtered image, where the extra planes that form the stacking fault are visible, and which are connected by ‘dislocations’.

**FIGURE 2:**
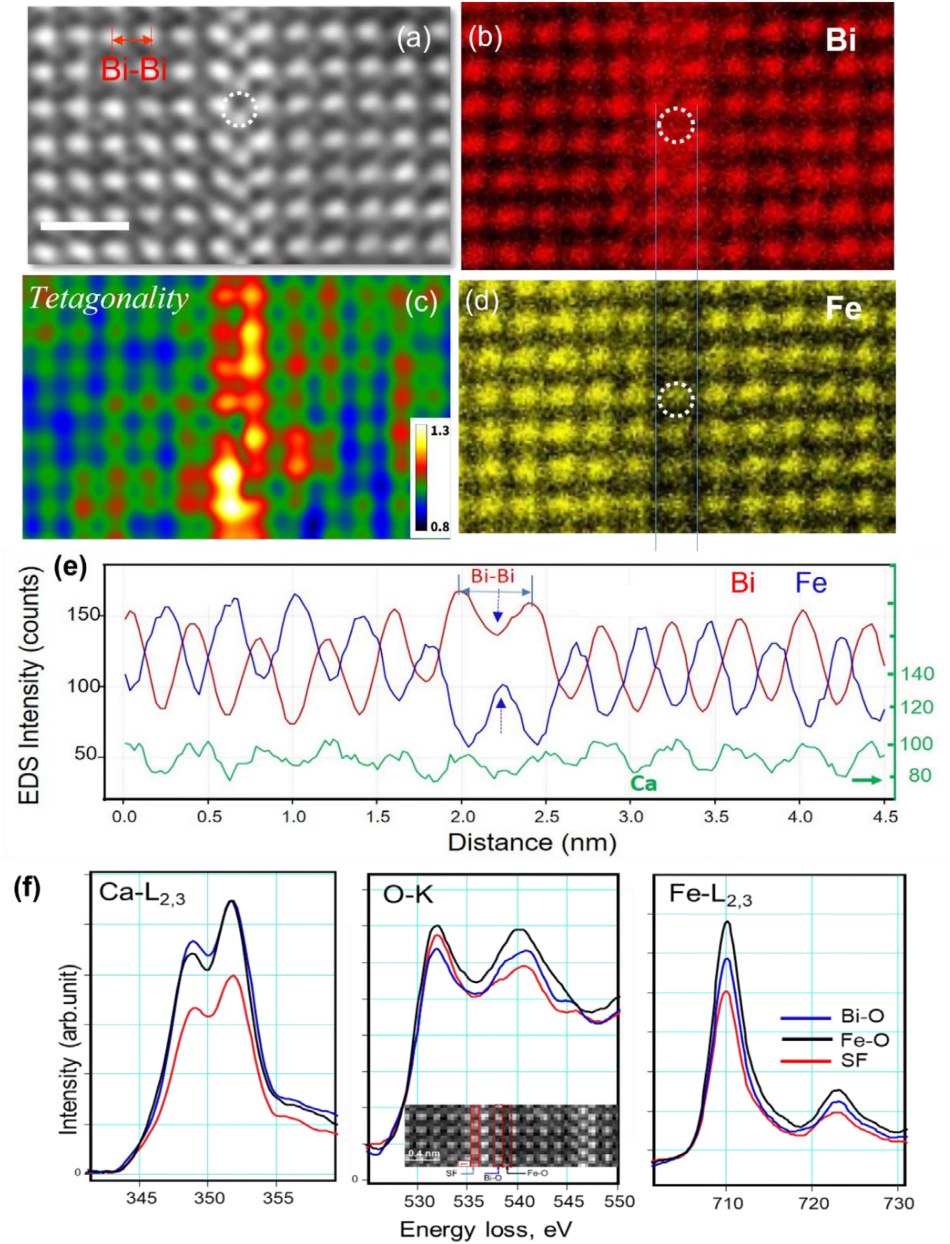
(a) HRTEM image of one faulted plane in the BFO matrix and corresponding atomic resolution elemental map (b) Bi, and (d) Fe, note that the Ca map is shown in the supplemental materials. The dotted circles indicate the same location. (c) The tetragonality map (the ratio of *a/b*). (e) profiles of Bi, Fe, and Ca elements were obtained by integrating the entire maps from top to bottom. The thin lines guide the positions of the Bi‐Bi atom columns. (f) Electronic structure analysis on one faulted plane (a portion of it is visible in the inset). ELNES of Ca‐L_2,3_, O–K, and Fe‐L_2,3_ edges. The fine structures were integrated over an area of 10 nm × 0.8 nm (as labeled by re frames) from the on/off‐faulted plane. Please note that the red curve (on the faulted plane), the blue curve (off the faulted plane, on the Bi─O plane with 2 atomic layers away from the faulted plane), and the black curve (off the faulted plane, on the Fe─O plane, with 4 atomic layers away from the faulted plane, Fe‐L_2,3_ signals are thus higher). The fine structures differ on the Bi─O and Fe─O planes of BFO. A STEM‐HAADF image (inset) indicates the measured locations of the faulted plane, Bi─O, and Fe─O planes.

To further ascertain the exact atom species or chemical composition at the planar defects, atom‐resolved elemental mapping (EDXS) was acquired. Figure 2 presents an atomic‐resolution STEM‐HAADF image of one faulted plane and corresponding elemental maps of Fe and Bi atom distributions (Figure 2b,d). The Ca map is shown in Figure S1. Elemental line profiles obtained by integrating over the entire maps (from top to bottom) are presented below (Figure 2e). As expected, Ca dopant atoms substitute for Bi atoms at identical lattice positions [17]. However, a close comparison of these maps reveals that some Bi atoms occupy Fe lattice sites by substitutional defects, which also agrees with the STEM contrast variations in Figure 2a. The elemental profiles (Figure 2e): 1) exhibit that the Bi intensities of two layers neighboring to the defective layer is higher than those in the nearby bulk; 2) at the defective layer, the Bi intensity at the Fe column is almost the same level as that of Fe columns at nearby bulk, while Fe atom intensity at the defect is significantly lower than that of Fe atom columns nearly (as labeled). Away from defects, the variations in Bi and Fe intensities are nearly complementary. Chemical analysis further confirms that Bi partially substitutes for Fe at the faulted plane, and that the two neighboring layers are enriched in Bi relative to the BFO bulk. Neglecting the likely different atomic position arrangements in the specimen thickness direction, based on atomic resolution elemental analysis, a simplified picture is that a layer‐like ‘BiO’ localized structure is formed. The consequence of such a substitution leads to an apparent increase in Bi‐Bi atom distances up to 4.66 Å, higher than the bulk average Bi‐Bi atom spacing in perfect BFO, with nearly 15% increments (Figure S1). The increased spacing indicates that the tetragonality of the faulted planes differs from that of the films. Using the geometrical phase analysis (GPA), a tetragonality map (*a*/*b* ratio, where a is the plane distance perpendicular to the SF plane and b is parallel to the SF plane; Figure 2c) could be obtained, illustrating a distinct *a*/*b* ratio across the faulted plane. This tetragonality map suggests that the Bi substitution for Fe causes a localized lattice distortion within one or two unit cells. It is anticipated that this might influence the BFO performance to some extent.

Such faulted planes alter the electronic structure, as revealed by electron energy‐loss spectroscopy (EELS) analysis. Figure S2 presents the surface plasmon map and integrated profile, revealing a variation between the defective and neighboring regions, with changes from 18.8 to 19.1 eV, most likely due to local modifications in electron density upon Bi replacement for Fe in the defective zones. However, the energy‐loss near‐edge structure (ELNES) in the defective regions presents a clear difference (Figure 2f). Using atomic‐resolved EELS mapping, we integrate the spectra over a framed region in the image (inset in Figure 2f), and the averaged fine structure of the Ca‐L_2,3_, O–K, and Fe‐L_2,3_ edges is displayed below for comparison. This enables probing the fine structure, even in a single Bi‐O row (blue curve) or Fe─O row (black curve) in the perfect BFO bulk, and a detectable change is observed in individual layers. Notably, the fine structure from the SF region (red curve), i.e., Ca‐L_23_, O–K, Fe‐L_2,3_ edges, is distinct from those obtained in nearby bulk, particularly for the O–K edge, where the second peak is largely suppressed. In addition, the white‐line ratios at SFs, Bi─O, and Fe─O atomic rows are different due to the localized electronic structure variations caused by the faulted stacking.

### 2.2 The Dynamics by In Situ Atomic‐Scale Observation

**FIGURE 3:**
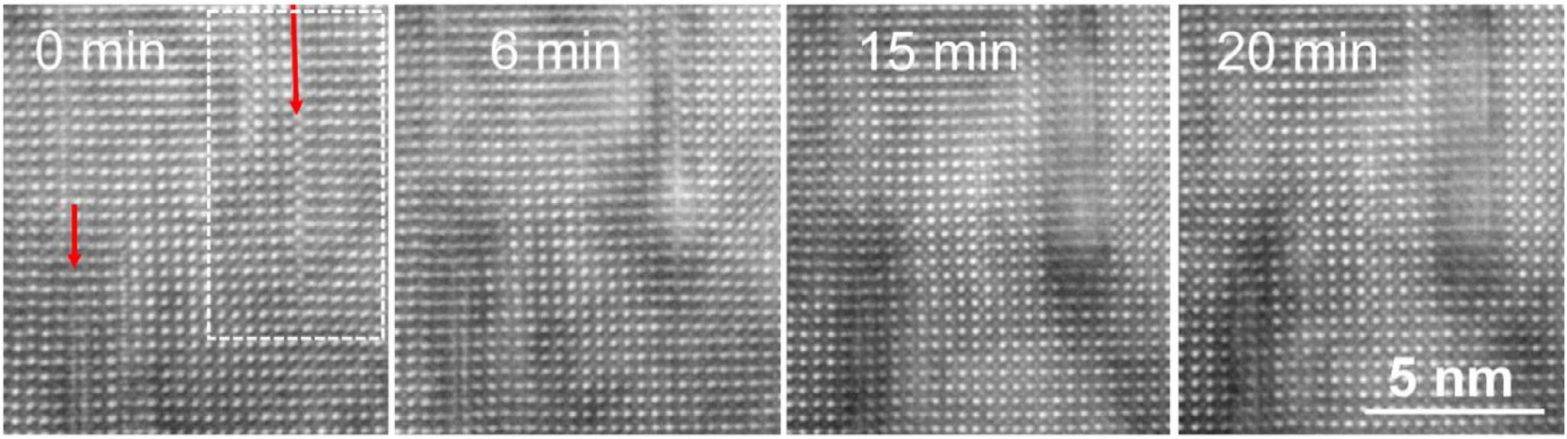
Time sequences of consecutive HAADF‐STEM images showing the amorphization process (here, only 4 images with irradiation periods of 0, 6, 15, 20 min are displayed), and arrows indicate one stacking fault and one dislocation (the lower one). Corresponding colored maps (from the dotted area including one faulted plane, as exemplified in the first image) are shown in Figure S4b, and the intensity changes in the disordered zone indicate that the amorphous area extends with irradiation.

The dynamic behavior of the single‐atom‐layer SFs under the electron beam is explored. Figure 3 shows the evolution of the SF under beam irradiation. After a few minutes of electron irradiation (i.e., 3–4 min) in STEM mode, the defective structure gradually evolved into a blurred contrast region with disordered, amorphous features atop the faulted planes, resembling premelting. The compositional analysis reveals that the features are amorphous Bi particles (an elemental map is provided after extended irradiation; see Figure S3). The electron beam triggers the formation of Bi nanoparticles. Continuous HR‐STEM images were recorded as a time series to precisely identify the sites and times at which the amorphous phase was initiated. Based on these images, together with other experimental images, one observes that the SFs and dislocations (labeled by an arrow, more visible in the filtered images Figure S4a) are actual initial sites where the premelting preferentially occurs and the blurred contrast appears (atom columns become blurred), indicative of Bi atom agglomerates atop of the faulted plane and becomes more. Such an amorphous phase can clearly be visualized when the filter is applied. The Bragg‐filtered colored images (Figure S4b), corresponding to the respective STEM images, show intensity changes around the SF over time. Compared with the initial state, the intensity in the filtered image decreases over time (indicating weaker contrast), suggesting that the amorphous phase accumulates atop the defects. The larger the reduced‐intensity region at the faulted planes, the more amorphous phase has formed. Using these time‐sequence images, the approximate coverage of amorphous phase zones can be determined, enabling visualization of the dynamics of amorphous phase formation. Experiments also show that such a premelting in STEM mode typically occurs at a critical current density, and the process is largely delayed or does not start when the probe current is below this threshold.

**FIGURE 4:**
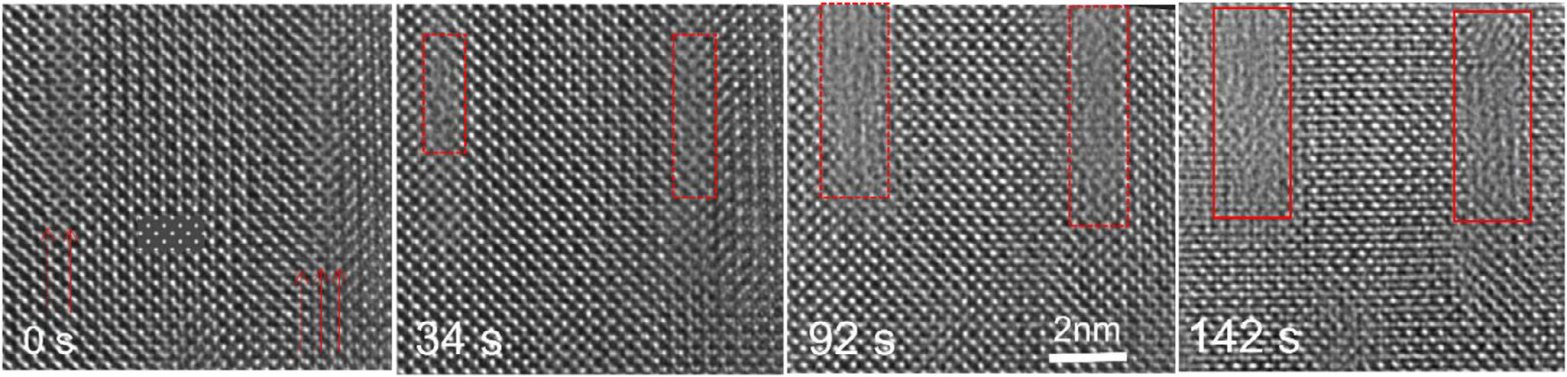
Time sequences of HRTEM images covering stacking faults show the structural evolution from 0 s to 142 s (the image at 6 s is not shown). Amorphization is initiated from the faulted planes (labeled with frames) after irradiation for a few seconds, and the disordered zone increases rapidly with time. One clipping of simulated images (under conditions: defocus of 1.7–3.7 nm and thickness of 31–55 nm) is inserted to confirm the bright contrast in the image originating from atomic columns. The arrows in the first image roughly indicate the defect's position. Careful examination shows that the imaging condition at the center of the image varies slightly with recording time due to a local deviation of 1 °‐2 ° from the zone axis beneath the electron beam. However, the amorphous zone produced is recognized and continues to grow (as indicated by the red frames).

However, the preferential premelting process at faulted planes can be significantly enhanced when the planes are exposed to an intense electron beam, e.g., a parallel beam in HRTEM mode. Figure 4 displays a real‐time series of in situ atomic‐resolution images obtained with a fast CCD, i.e., at 0, 34, 92, and 134 s. From image simulations, the sample thickness is estimated to be around 35–45 nm, and the recorded HRTEM images confirm that the bright contrast corresponds to atomic columns under experimental conditions (clipping of simulated images overlaps). Two SF regions (as framed) are covered, in which atomic planes are distorted at faulted planes. Based on experimental observations, Bi atoms are rapidly expelled from the defect region only after a few seconds of irradiation (6.0 s; not shown here). The amorphous region becomes visible only after 34.0 s. At 142s, the amorphous liquid region extends rapidly and covers several atomic planes. Compared with observations in STEM mode (convergent probe), the process is significantly accelerated in HRTEM mode (parallel, intense beam). Close examination of atomic‐structure images also revealed that premelting initiates at apparent defect sites and then spreads to nearby regions. Taken together, the faulted planes experienced less disordered, more disordered, less amorphous, and more amorphous states successively until a significant amorphous phase developed at the initial faulted planes. It should be mentioned that premelting results in liquid droplets on defects. However, such a small volume of liquid droplet can swiftly transform into a solid state under the beam. Therefore, here, liquid and solid states are all classified into ‘disordered’ or ‘amorphous’ zones.

**FIGURE 5:**
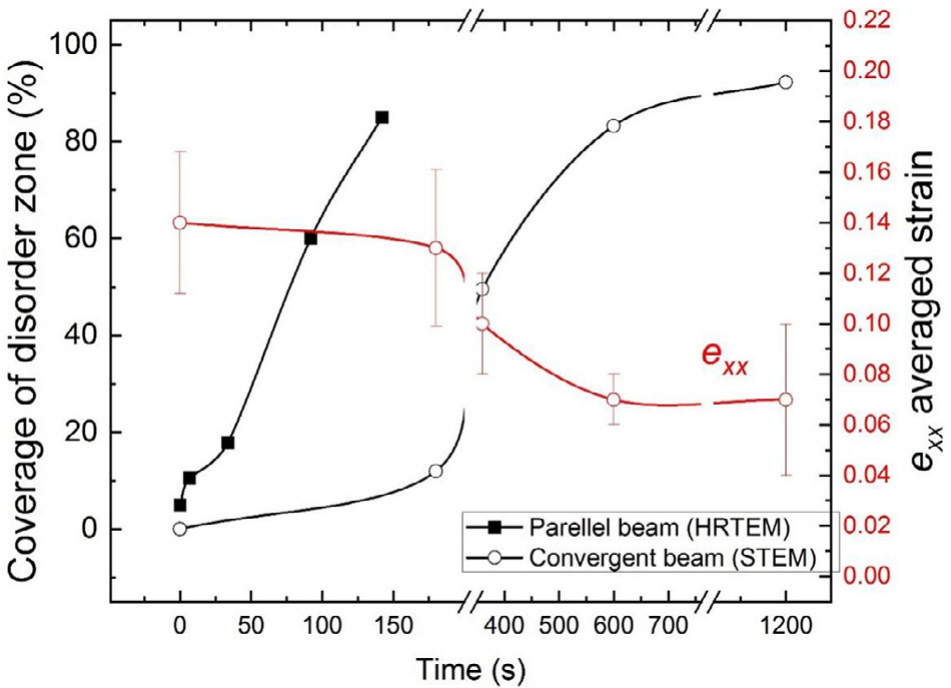
The dynamics of amorphization at planar defects under parallel (HRTEM) and convergent (STEM) illumination. It shows the relative rate of disordered zone formation, e.g., the coverage of the amorphous phase across the entire crystalline area within a defined area (4 × 13 unit‐cell area, covering one planar defect) over time. The red curve represents the local strain variations associated with the amorphization process (STEM mode). The strains were approximated by averaging the strain distribution over an area 6.25 nm long and 2‐unit cells wide (0.8 nm) in the *e_xx_* maps (obtained with GPA).

**FIGURE 6:**
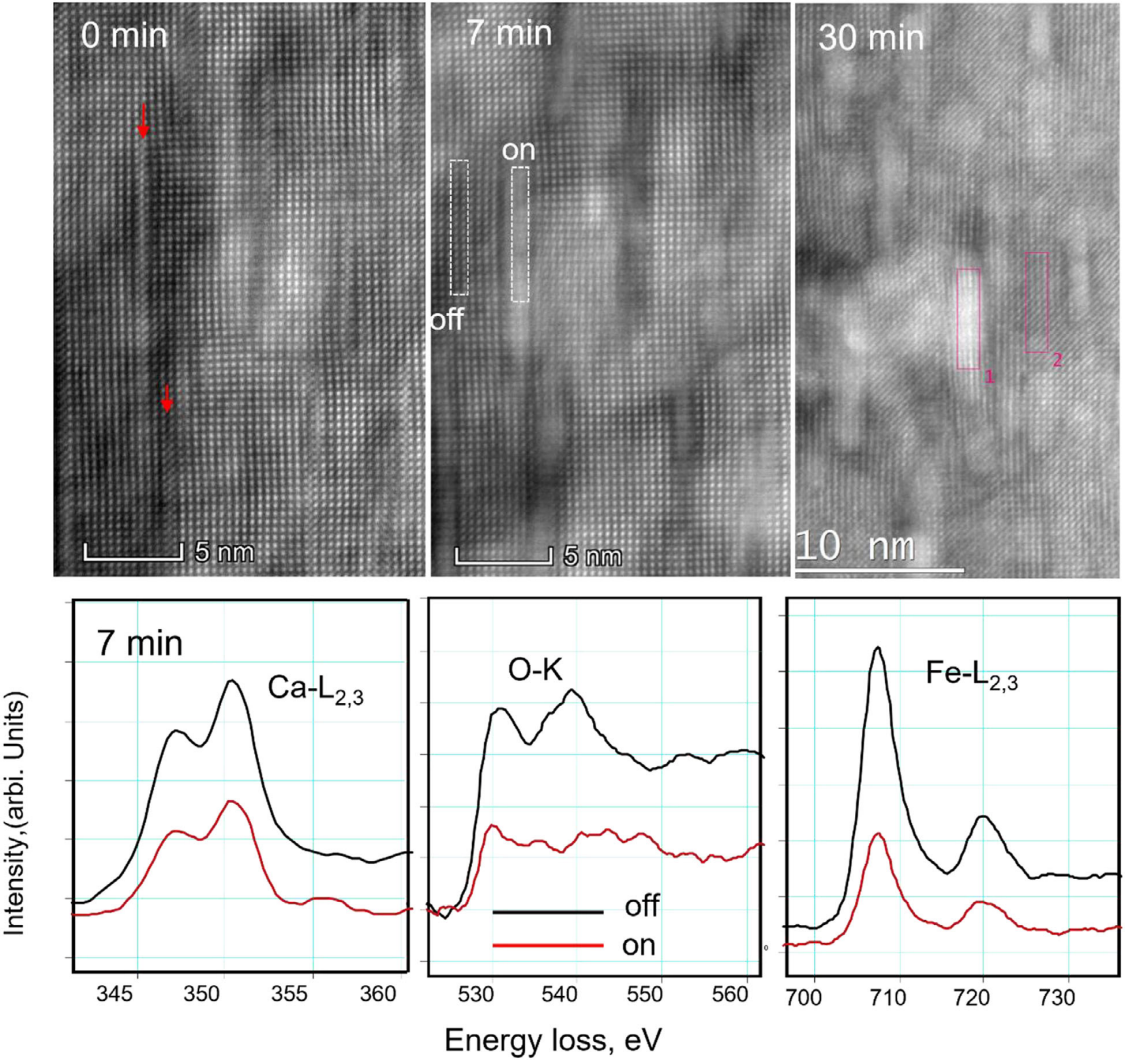
STEM‐HAADF images were recorded before and after 7 and 30‐min irradiations, and corresponding strain maps are shown in Figure S7. The lower panel shows variations in the electronic structure (the black and red curves correspond to the nearby bulk and the defect after 7 min of irradiation, respectively). The ELNES was obtained by integrating the area using a 10 nm × 0.8 nm frame (as labeled). The comparison indicates that after 7 min of irradiation, the O–K edge changes prominently, except for a reduction in the intensities of the Ca‐L_2,3_ and Fe‐L_2,3_ edges. Here, the O–K, Ca‐L_2,3_, and Fe‐L_2,3_ edges in the nearby bulk are averaged from the Bi─O and Fe─O rows, rather than from individual rows (as Figure 2f). STEM images were taken at a probe current of 200 pA and a dwell time of 500 ns.

The dynamics of the premelting process can be quantitatively evaluated by measuring amorphous‐phase coverage around the faulted plane (as described in the experimental section). The change in amorphous coverage over time is plotted in Figure 5. The local melting process (amorphous phase) is relatively faster in HRTEM than in STEM mode. The plot also shows that the amorphous‐phase probation is longer in STEM. The apparent growth of the amorphous phase is visible only after 20 min, whereas it occurs only within a few seconds under HRTEM mode. As with amorphous nucleation and growth, forming an amorphous phase takes longer with a less intense beam. The sequential STEM images in Figure 6 also verify that the emergence of the complete amorphous phase takes longer, up to 30 min.

The transient strain state can be approximated through geometrical phase analysis. The magnitude of averaged strains obtained over a specific area around a single stacking fault is shown (Figure S5) for comparison. The *e_xx_* strain maps represent in‐plane strain (perpendicular to the faulted plane). The strong, bright region (corresponding to a high‐strain state, here tensile strain) decreases with irradiation, while the out‐of‐plane strains do not change significantly. The magnitude of strain can be described by the average *e_xx_*, integrated over a region 6.25 nm long and 2‐unit cells wide (∼0.8 nm) at/around the stacking fault. The result is shown in Figure 5 (red curve), which demonstrates how local strain evolves over time at the unit‐cell scale. The local strain gradually vanishes as more amorphous phase forms. Based on this, we can state that localized melting at defects releases local lattice strain and will rearrange atoms.

### 2.3 The Atomic and Electronic Structure Change Caused by Premelting

Premelting at the SFs alters not only the atomic structure, but also the electronic structure. During premelting, an atomic‐scale transient process can be captured with a fast CCD. Thus, the local strains imposed on two neighboring atomic planes of the SF can be roughly assessed. For simplicity, the transient planar spacings were determined here. Figure S6 shows a linear relationship between the planar spacing (neighboring plane distance) and irradiation time, indicating a rapid change every 100 µs.

Concomitant with the formation of amorphous Bi nanoparticles, the local lattice structure alters accordingly. Except for this, the corresponding electronic structure varies. Figure 6 shows STEM‐HAADF images, with an apparent amorphous phase emerging atop the initial faulted region after 7 min of irradiation (local strains were evaluated in Figure S7). After 30 min, many more amorphous phases had formed, resulting in more blurred, brighter‐contrast regions. The lower panel presents the corresponding EELS spectra for the amorphous phase (obtained by integrating several spectra over the amorphous phase), i.e., ELNES variations of Fe‐L_2,3_, O–K, and Ca‐L_2,3_ before and after 7 min (pre‐melting has already started). Some differences are obviously visible. Firstly, the Fe‐L_2,3_ and Ca‐L_2,3_ edges exhibit a subtle difference in ELNES (as evidenced by the slight variation in white line ratios), which is comparable to that of bulk BFO. This is attributed to the formation of the Bi amorphous phase atop the faulted plane, while a certain amount of BFO remains beneath. Thus, hardly any changes in edge shape in Fe‐L_2,3_ and Ca‐L_2,3_, except for reduced intensity. However, the O‐K edge significantly varies after the formation of the amorphous phase. The O–K absorption edge is primarily suppressed, i.e., the two distinct peaks at ∼530 and ∼540 eV disappear. This is closely linked to the local O electronic‐state change after Bi is depleted from the parent lattice, which likely results in a distorted Fe─O octahedral. With prolonged illumination, the O–K absorption edges remain relatively unchanged. The comparison of current O–K ELNES (from the BFO matrix before illumination) to those in *a*‐Bi_2_O_3_ [22] exhibits a similar trend, e.g., two prominent peaks detectable at the energy of 530∼540 eV. However, the irradiated samples exhibit different features.

## 3 Discussions

Previous atomic‐scale HRTEM investigations of Bi droplets/phases have revealed interesting phenomena, including the role of defects (domain walls, grain boundaries, and dislocations) that can serve as initial sites for Bi crystal‐phase transformation from crystalline to liquid phases, as well as the effects of heating [19, 21, 23, 24]. It clearly shows the critical conditions and governing parameters under which droplet transformation (melting or crystallization) occurs, as well as the mechanism of the transformation. Recently, it has also been demonstrated that beyond crystal size, coalescence growth dynamics involving twins are highly dependent on crystal defect density and the approach pathways of the crystals. Despite these findings, however, it is not yet clear how and where those Bi droplets (or nanoparticles) form (or detach) from the parent lattice, nor what the underlying mechanisms are. In contrast, our current research sheds light on the topic by clarifying one of the mechanisms that drives Bi atoms out of the parent BFO lattice, particularly at the faulted planes.

**FIGURE 7:**
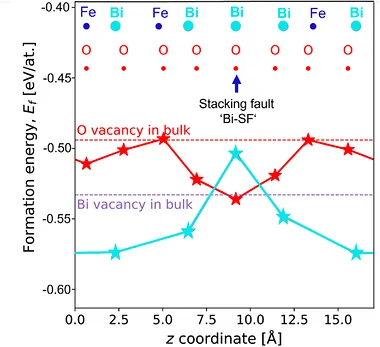
The formation energy (*E_f_*) of the Bi vacancy and the O vacancy at the stacking fault of BiFeO_3_ and nearby using DFT calculations. Straight dotted lines are the *E_f_* of the Bi vacancy and the O vacancy in bulk BiFeO_3_. The calculated results show the interaction energy between the Bi vacancy and the Bi‐faulted plane, as well as with the nearby bulk layers (i.e., the first Bi─O layer (next to the SF) and the second Bi─O layer away from the SF). The plot shows the interaction energy distribution, indicating that Bi‐Vac repel each other much more strongly (or are less stable) in the faulted plane than in the nearby Bi─O layers. The preferred configuration is one in which the Bi vacancy is not directly at the SF (*E_f_* = −0.585 eV/at.). The *E_F_* change for the O vacancy in bulk BFO differs from that for the Bi vacancy. In contrast to the Bi vacancy, which is repelled by SF, oxygen vacancies are more strongly attracted to it, as the O vacancy at the stacking fault position has a lower energy than the Bi vacancy.

The most intriguing finding in this work is that premelting preferentially initiates at defective sites of the parent BFO lattice. To rationalize this, DFT calculations were performed. The Bi vacancy (abbreviated as ‘Bi‐Vac’) formation energies in the stacking faulted plane and the bulk were calculated (see Supplementary Materials). The interaction energy between Bi‐Vac and SFs, or between Bi‐Vac and bulk, is derived and plotted in Figure 7. For comparison, the oxygen‐vacancy interaction with SFs or the bulk is also superimposed. From it, what we can quickly conclude: **(1)** Bi vacancies are energetically preferred to O vacancies (as vacancy formation energy is lower for Bi than for O); **(2)** Bi vacancies are repelled by the ‘Bi‐SF’ (a Bi plane replacing the Fe plane in bulk), but are attracted from the bulk to the nearby SF region. Conversely, **(3)** O vacancies appear to be attracted (or segregated) to the SF. Furthermore, these calculations indicate that creating a Bi atom vacancy is energetically much easier in BFO bulk than creating an O atom vacancy.

### 3.1 The Bi Vacancy Effect on Premelting

It indicates that the interaction energy of faulted planes with Bi‐Vac is higher than that of neighboring planes with Bi‐vacancy (although a slight variation in bulk). It discloses that the faulted planes and Bi‐vacancy repel each other energetically. This suggests that forming Bi vacancies at the faulted planes is more difficult than in bulk, which also means that Bi atoms are hard to detach from the lattice. This seems contradictory to experimental observations. However, it is worth noting that the calculations were conducted without accounting for strain.

Given that Bi atoms replacing Fe at the faulted plane cause the plane distance expansion (Figure 2S), such a faulted plane (usually, one or two atom layers) is compressively strained with regard to the neighboring BiO planes that received tensile strain. That means creating a faulted plane imposes tensile stress on neighboring BFO films, while the faulted plane itself experiences compressive strain from its surroundings. Considering the effect of compressive strain, the calculated results are shown in the Supplementary Materials. The formation energy required to create a faulted plane in BFO bulk (‘Bi‐SF’) rises, i.e., from −0.531 (unstrained) to – 0.480 eV (strained), which suggests that SF becomes even more unstable under compressive strain. This means that the strained SF region is not preferred. The energies for those SFs with a Bi‐vacancy located on different Bi‐O planes also indicate that the preferred configurations are still at the neighboring planes rather than the faulted plane.

On the other hand, it is noted that the Bi‐vacancy formation energy is compressive strain‐dependent in BiFeO_3_ [25]. This indicates that compressive strains could trigger a remarkable reduction in the formation energy of Bi‐vacancies. On this basis, we rationalize our observation that Bi atoms are easily “squeezed” out of the faulted planes, energetically and unfavorably residing in the compressively strained lattice. The energy difference between moving Bi atoms in the lattice and in the defective zone serves as a driving force. When stimulated by an external beam and by compressive strain simultaneously, Bi atoms at faulted planes are more readily displaced from their positions than those in the surrounding regions, thereby relieving local strain. As shown below, together with numerous additional O vacancies continuing to aggregate at the faulted planes, the amorphization process gradually accelerates with increasing irradiation.

### 3.2 The O Vacancy Effect on Premelting

Our DFT results show that the formation energy of the O vacancy is higher than that of the Bi vacancy in BFO (Figure 7). However, the O vacancy could be present in the BFO films, especially when doped with Ca [26], forming an ion conductor. The O vacancy is expected to influence the behavior of the Bi atom. The presence of O vacancies modifies the coordinates around Bi atoms, changes the octahedral, and induces severe lattice distortions. As a consequence, it leads to a more disordered structure. However, this is a very complicated scenario because the oxygen atom contrast is not visible. It is hard to separate its effect from the experimental observations and its contribution to the amorphization process. Nevertheless, based on our DFT results, a simple understanding of the role of the O vacancy during premelting could be proposed. Energetically, O vacancies will readily be attracted to defects, such as stacking faults, and such a process could be largely enhanced when irradiated by electrons. The longer the irradiation period, the more O vacancies will aggregate or be attracted to an SF (see the irradiation analysis below). The greater the accumulation of O vacancies at SFs, the more disordered the structure will be. **The O vacancy accelerates the amorphization process**. It also explains why no noticeable change in the nearby bulk BFO was observed during irradiation. Taken together, the amorphous phase formation due to premelting is enhanced by the possible presence of O vacancies.

**FIGURE 8:**
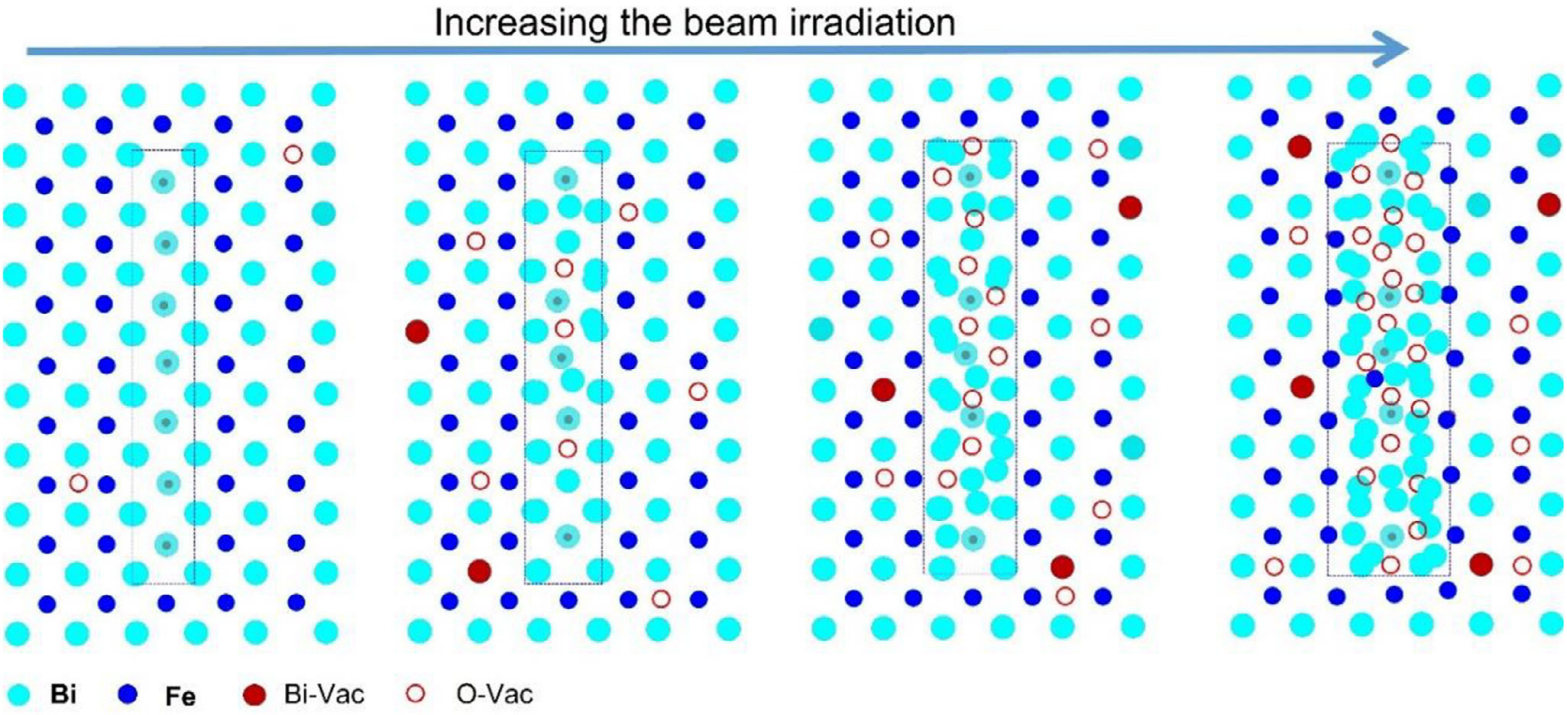
Schematic illustration of the amorphization process initiated at a faulted plane (labeled by frames) through an atomic model over time. With increasing irradiation, Bi atoms are displaced from the fault planes, while the concentration of O vacancies increases and accumulates there, leading to continued structural disorder and, eventually, the formation of an amorphous region around the initial faulted plane. Note that, for clarification, the original oxygen atom columns are not shown in the atomic models.

Such a premelting dynamic process could be schematically depicted by an atomic model that covers one SF (Figure 8). Bi atoms migrate from the lattice at the faulted plane, driven by an energy threshold. Compared to the neighboring bulk BFO, atoms at the faulted planes become more active and mobile once stimulated by an electron beam. Structurally, the strain release in bulk BFO effectively minimizes the total energy (including strain energy). With the influence of the O vacancy, the lattice distortion is significantly enhanced. Consequently, disordered Bi atoms beneath and near the faulted planes migrate to the specimen surface, rapidly forming a nanosized amorphous phase, as experimentally observed in Figures 3, 4, and 6.

The preferential melting (or ultrafast amorphization) at defects has been reported in metals [27]. Unlike in current oxides, molecular dynamics simulations of metals such as Cu have shown that stacking faults induce a negligible reduction in melting temperature but a slight increase in nucleation rate and nucleation probability [28]. A ‘virtual melting’ may occur at temperatures well below the melting temperature under high strain rates [29]. The preferential premelting observed here, i.e., forming an amorphous phase, is somewhat similar to the above work if significant mismatch strain is considered at faulted planes. Accompanied by premelting, the localized strains are released. Furthermore, given the stringent growth conditions for stoichiometric BFO, planar defects could inadvertently be incorporated during deposition. These defect regions could serve as initial sites for the formation of an amorphous phase under the electron beam, creating ‘bright‐contrast’ patterns throughout the films [18].

### 3.3 On Electron Beam Irradiation

To gain insight into the amorphization process and have some clues about the quantitative effect of the electron beam on the amorphization process of the faulted planes, we intentionally irradiate the stacking faults with 200 and 300 kV electron beam energy under different conditions. The experiments reveal that the observed planar defects remain stable under 200 kV electron irradiation for tens of minutes, possibly longer. Even when the beam dose is increased, the planar defects do not readily transition to the amorphous phase, and no obvious premelting occurs at faulted planes. A time sequence of HRTEM images, cut from a single video recorded at a beam current of 202 pA/cm^2^, is shown in Figure S8 to demonstrate the stability of planar defects under 200 kV irradiation. However, under irradiation with 300 kV electrons, planar defects could readily evolve into amorphous phases after a short exposure. The premelting could occur rapidly under parallel illumination (HRTEM mode). Increasing (or decreasing) the flux reduces (or prolongs) the time to amorphization, indicating a flux dependence of amorphization under 300 kV, with an approximate inverse relationship.

When an electron beam illuminates the material, incident electrons transfer energy to the atoms that comprise the material [30]. High‐energy electrons can damage materials through ionization and displacement damage, and even heating [31, 32, 33, 34, 35]. It is found that the beam damage at 300 kV in BFO is most likely ascribed to atomic displacement damage [36]. When the transferred energy provided by the incident beam is greater than the threshold displacement energy (*E_d_*) for each constituent atom, point defects in the materials are created by suffering the knock‐on damage. The transfer energy (*T_m_*) provided by incident electrons could be calculated using the following formula: *T_m_ = 2E(E + 2m_0_c^2^)/Mc^2^*, where *E* is the energy of the incident electron, *m_0_* is the rest mass of the electron, *c* is the velocity of light, and *M is* the mass of the displaced atom. The calculated transfer energies (*T_m_*) for incident beams of 200 keV and 300 kV to Bi atoms are 2.5 and 4.1 eV, to Fe atoms are 9.4 and 15.2 eV, and to O atoms are 19.1 and 53.1 eV, respectively. For BFO, the exact experimental and theoretical threshold energy (*E_d_*) required to create knock‐on damage and generate stable point defects remains unknown. However, ∼ 25 eV was proposed as a general guideline of threshold displacement energy [37]. Molecular dynamics simulations were used to calculate the threshold displacement energies (*E_d_*) for each atom type in BaTiO_3_ perovskite [38]. The obtained values are approximately 40 eV for O, 64 eV for Ba, and 97 eV for Ti atoms, which are comparable to ab initio‐calculated and experimentally derived values in perovskites. This also provides a reference for the knock‐on damage in BFO.

As seen, the *T_m_* values for Bi, Fe (at 200 and 300 kV), and O atoms at 200 kV are below the threshold energy *E_d._* Clearly, the transferred energy to O atoms with 300 kV electrons irradiation is greater than the generally considered threshold energy *E_d_* for atomic displacement, i.e., 25 or 40 eV (reference value in BaTiO_3_). This indicates that knocking atoms off the sample exit surface through the displacement damage in BiFeO_3_ is not significant at 200 kV. This also explains why no obvious amorphization was observed under 200‐kV electron irradiation.

The calculated transferred energy (*T_m_*) from the incident 300 keV beam indicates that more O atoms can be easily displaced from the sample's exit surface by the electron beam (displacement damage), namely, the accumulation of O vacancies, similar to the observations in the case of Bi_2_WO_6_ [39]. Furthermore, our DFT calculations indicate that O vacancies are energetically attracted to stacking faults. This implies that, with continued irradiation, more oxygen vacancies will accumulate preferentially at faulted planes, leading to a more localized, disordered structure and thereby accelerating the amorphization process. Thus, we can state that beam irradiation with 300 kV electrons accelerates amorphization by attracting numerous O vacancies created by irradiation (or doping) and releasing local strains.

## 4 Conclusions

(1) The as‐deposited defects in BFO have been identified to be stacking faults with a single‐atom layer non‐stoichiometric phase, which could be the preferential sites that the amorphous phases initially nucleate. 2) Both experiments and theoretical calculations corroborate that the premelting phenomenon was essentially triggered by local compressive strains on the faulted planes imposed by the surrounding matrix. It results from the release of local strains in response to an external stimulus (i.e., an electron beam). Moreover, oxygen vacancy accumulation induced by knock‐on damage may significantly accelerate the amorphization process at faulted planes. The synergistic effect alters the dynamics of amorphous phase formation. 3) Accordingly, the local atomic structure evolves to a disordered phase. Corresponding electronic structure experiences an alternation resulting from amorphous phase formation, which is reflected in the slight change of O–K and Fe‐L_2,3_ absorption edges.

## 5 Experimental Section

### 5.1 Thin Film Deposition

A Ca‐doped Bi_0.9_Ca_0.1_FeO_3_ film was fabricated via pulsed laser deposition (PLD). The thin films were deposited on a single‐crystalline (100) oriented SrTiO_3_ substrate (Kejing Materials Technology Co. Ltd., China) by PLD technique using a KrF excimer laser (Lambda Physik COMPEX PRO 205F, λ = 248 nm) as the ablation source with a pulse repetition rate of 5 Hz. The laser beam energy was fixed at 320 mJ/pulse for the Ca‐doped films. Before film deposition, the substrates were ultrasonically cleaned with acetone, alcohol, and pure water, and then blown dry with high‐purity nitrogen. Afterward, they were immediately loaded into the PLD chamber. The vacuum chamber was evacuated to approximately 10^−4^ Pa. During film deposition, the substrate was held at 700°C, and the oxygen pressure was set to 3.0 Pa. The total deposition time for both film samples was 30 min, resulting in a thickness of approximately 50 nm for the Ca‐doped film. The crystalline structure of the films was investigated using high‐resolution x‐ray diffraction (HRXRD) with a four‐circle single‐crystal diffractometer (D8 Discover, Bruker, Germany) and a Cu Kα1 monochromatic radiation source (λ  =  1.5406 Å).

### 5.2 TEM Characterization and Irradiation Tests

#### 5.2.1 TEM Specimen Preparation

Cross‐sectional TEM specimens were prepared using standard TEM sample‐preparation techniques, including grinding, polishing, and dimpling. Ar+ ion milling was carried out at 4.0 kV with an angle of 6°, followed by a final low‐voltage ion‐milling of 2.5 kV with an angle of 2°. Our experiments were performed on several microscopes for different purposes.

#### 5.2.2 Characterization

High‐resolution TEM and STEM investigations were performed in an FEI Titan Themis 60–300 cubed microscope operated at 300 kV. This instrument is equipped with an image‐side spherical aberration (Cs) corrector (CEOS GmbH). For HRTEM imaging, the C_S_ value was set to −3.0 µm. Images were recorded with a fast 4 K × 4 K 16‐bit CMOS‐based CETA camera at a frame rate of ∼500 ms. For STEM ADF imaging, the spherical aberration constant of the probe‐forming lens is C_S_ = 1.2 mm. To achieve the best resolution, an optimal convergence angle of 9.6 mrad was obtained with a 50 µm aperture and a spot size of 9. A camera length of 73 mm was set, and the ADF detector's collection angle range is approximately 79.5–200 mrad. The probe current was tuned to about 30 pA. To achieve a better signal‐to‐noise ratio (S/N), Drift‐Corrected Frame Imaging was used to average multiple frames.

For analytical work, a 300 kV cold‐field emission TEM (JEOL ARM300F) equipped with double C_S_‐correctors was also used in this study to obtain atomic‐resolution elemental maps. Two windowless energy‐dispersive x‐ray spectroscopy (EDXS) detectors, each with an active area of 100 mm^2^, are mounted on the microscope, which is positioned very close to the specimen, providing a high solid angle (1.7 sr). A Quantum filter (Gatan) is equipped with a dual EELS capability. The spectrum image (SI) and the ADF signal in the STEM‐EELS experiments were acquired simultaneously at a beam current of ∼50 pA. EELS spectrum images were taken under the following conditions: collection and convergence semi‐angle (mrad): 20.5 and 19.6, respectively. Energy resolution: Energy range: 0–350 eV for low‐loss spectrum and 300–812 eV for core‐loss spectrum; dispersion: 0.25 eV/channel; exposure time: 0.01s for individual core‐loss and low‐loss spectra. A low‐loss map was constructed after subtracting the background and fitting to the peak center.

To visualize fine‐structure variations, we integrate multiple spectra over a specific range to improve the signal‐to‐noise ratio. Further, for the respective EELS spectrum acquired on amorphous nanoparticles and nearby bulk, we also applied an ‘area‐method’ to cover the region of interest, i.e., 10 nm × 0.8 nm. After background subtraction, these spectra were smoothed with a 2‐channel low‐pass filter.

Before conducting quantitative analysis, all HRTEM images were calibrated. To better visualize the local strain, an approach similar to that of geometrical phase analysis [17] was applied, selecting more than two reflections (weighted averages of multiple reflections, as described in reference [14]) to increase the signal‐to‐noise ratio in the phase images. A Gaussian mask of size **g**_100_/3 was placed around the reflections. Image simulation (using JEMS software) was performed to confirm the experimental images, thereby excluding image artifacts that may have occurred during observations. The simulation conditions were as follows: 300 kV, a C_S_ value of 1 µm, and an orientation along the [100] zone axes. A thick‐focus map was calculated for comparison with images taken under experimental defocus.

#### 5.2.3 Irradiation Tests

HRTEM irradiation experiments were recorded as a series of consecutive images, each with a 0.33 ms interval, at an image size of 19.4 nm × 19.4 nm and a pixel size of 0.019 nm × 0.019 nm. The beam current is **∼**50 pA. Under such conditions, the total irradiation time for the entire imaged area can reach 2.5 min. The irradiation experiments in STEM mode were conducted under the following conditions: probe current, 30 pA; pixel dwell time for image acquisition, 200 ns. The irradiated area is about 21 nm × 21 nm. Under such conditions, the total irradiation time for the entire imaged area can reach 20 min.

Except for the 300 kV Titan microscope, beam irradiation tests were also conducted on a JEOL 2100F at 200 kV, equipped with an image‐side spherical aberration (C_S_) corrector, which demonstrated a resolution of 1.2 Å at 200 kV. The aberration coefficient is set to a value close to 1 µm, under which the HRTEM images were recorded with slightly over‐focused conditions. A CCD Orius camera is used to acquire a sequence of HRTEM images with a pixel size of 2048 × 1336. The current density ranges from 85 to 202 pA/cm^2^.

#### 5.2.4 Dynamic Determination During Irradiation

Under the beam, a liquid‐to‐solid transformation can occur, a phenomenon that is difficult to capture in a very small region. During irradiation, Bi nanoparticles can emerge in liquid form or rapidly transform into the solid state. Both states were observed experimentally. To track the dynamics, a ‘disordered’ region (including liquid droplets and amorphous areas) around the planar defect is defined as the amorphous‐phase coverage and is measured over time.

### 5.3 DFT Calculation Approach

The original configuration of BFO corresponded to a ferromagnetic (FM) configuration. Since the FM‐BFO lies significantly above the energy convex hull (0.255 eV/at.), an anti‐ferromagnetic (AFM) configuration (0.012 eV/at. above the convex hull) was also tested. The lattice constants for FM‐BFO are: ***a*** [Å] 5.58, ***b*** [Å] 5.58, ***c*** [Å] 7.89. The energetics were calculated using density functional theory (DFT), and the computational details are as follows.

#### 5.3.1 Formation Energy (E_f_) of Stacking Fault

A summary of calculations with one Fe atom in one plane (per simulation box) is changed to Bi atoms. The calculation was performed in a corresponding supercell (1 × 1 × 3) based on a unit cell containing 10 (FM) or 20 (AFM) atoms. The result is shown in Table S1.

#### 5.3.2 Formation Energy (E_f_) of Vacancies

Next, we look at Bi vacancies in perfect BFO (without stacking faults). Vacancy concentration, ***x_vac_***, is calculated as ***x_vac_*** = (1/(***N_at_*** + 1), where ***N_at_*** is the number of atoms in the supercell containing a Bi vacancy or O vacancy. Suffix FR refers to fully relaxed calculations (including cell shape); other calculations keep the ***a*** and ***b*** (in‐plane) lattice parameters fixed to those of bulk structures and only relax the out‐of‐plane spacing ***c***. Atom positions are relaxed in all cases. The result is shown in Table S2.

#### 5.3.3 Vacancy‐SF Interaction Energies

The interaction energies are defined as:

$$E_{int}=E\left(perf\right)+E\left(SF+vac\right)-E\left(SF\right)-E\left(vac\right),$$

where each of the energy terms above the total energy of equivalent supercells, i.e., the number of atoms of ***E*** (perf) and ***E*** (SF) are the same and contain one more atom than the cases corresponding to ***E*** (SF + vac) and ***E*** (vac). There was always one vacancy per plane; the supercells were 2 × 2 × 2 or 2 × 2 × 3, yielding 80 and 120 atoms in the perfect FM case. The interaction energy as a function of the plane containing the Bi vacancy is shown in Figure 7 (with a case of 2 × 2 × 2 (FM)).

#### 5.3.4 Vacancy‐SF Interaction Energies Under Strain

The Bi vacancy formation energy*, E_f_* [eV/at], is calculated for 2 × 2 × 2 supercells with compressive‐strained and unstrained structures. The atomic model on the right shows the position of the Bi vacancy and the faulted plane. The energy required to create one Bi vacancy (labeled by “Bi‐Vac”) at SFs and nearby Bi─O planes (the first and second Bi─O layers defined as ‘1Bi’, ‘2Bi’, respectively, referred to the schematic figure in the DFT method in the supplementary materials). The energy required to form one faulted plane by substituting one Bi site for one Fe site is also listed for comparison. The SF formation energy is high due to the considerable strain (compression along c) at the faulted plane position. A lot of strain can be relieved by the vacancy, so it is always preferred to bring the vacancy in the vicinity of the SF (*E_f_* < −0.527 eV/at.) rather than to have a strained SF region (*Ef* = −0.480 eV/at.) and a far isolated vacancy (*E_f_* = – 0.533 eV/at.). The result is shown in Table S3.

## Conflicts of Interest

The authors declare no conflicts of interest.

## Supporting information




**Supporting File**: smll74384‐sup‐0001‐SuppMat.docx.

## Data Availability

The data that support the findings of this study are available on request from the corresponding author. The data are not publicly available due to privacy or ethical restrictions.
