# Supplementary material for: In Situ Atomic‐Scale Observation of Preferential Premelting at Oxide Crystal Defects
Source: Small. 2026 Jul 1;22(47):e74384. doi: 10.1002/smll.74384 (PMC13495802; doi:10.1002/smll.74384)
Supplement: Supplementary file 1 [file SMLL-22-e74384-s001.docx]

**Supplementary materials**

**In situ atomic-scale observation of preferential premelting at oxide crystal defects**

Zaoli Zhang^1^, Zhuo Chen^1^, Yong Huang^1^, David Holec^2^, Yonghui Zheng^3^, Jan Michalička^4^

^1^Erich Schmid Institute of Materials Science, Austrian Academy of Sciences, A-8700 Leoben, Austria

^2^ Department of Materials Science, Montanuniversität Leoben, A-8700 Leoben, Austria

^3^Key Laboratory of Polar Materials and Devices (MOE), Department of Electronics, East China Normal University, Shanghai 200241, China.

^4^ CEITEC Nano, Brno University of Technology, Purkyňova 656/123, 612 00 Brno, Czech Republic

Correspondence: zaoli.zhang@oeaw.ac.at

**Keywords:** in-situ, atomic structure, EELS, BiFeO₃, defects, DFT, STEM

**Fig. S1** HAADF – STEM image and corresponding Ca elemental map and Bi-Bi atom column distance.

**Fig. S2**	HAADF-STEM image (the same as the inset in Fig. 2f), the corresponding surface plasmon distribution (middle color image, low loss map). Please note that plasmon variations occur when crossing the individual SF, Bi-O, and Fe-O layers. Here, the Pseudo-Voigt 1 function was used to fit each plasmon peak in the individual low-loss spectrum, thereby determining the peak centers, and the corresponding plasmon map was obtained from these peak centers. The line profile beneath was acquired by integrating the plasmon map from top to bottom over the entire area

**Fig. S3** HAADF –STEM images and elemental distributions of Bi, Fe, and O atoms after an extensive irradiation. Apparently, larger Bi nanoparticles have been formed on the surface, where the other atom species are depleted.

**Fig. S4** (a) A series of Bragg-filtered images (0 min, 6min, 15min, 20 min) demonstrating the dislocation (labeled) evolution at the stacking fault with the irradiation. Inserts are the color maps at the stacking fault. (b) The Bragg-filtered colored images**,** corresponding to the respective STEM images in Figure 4, show intensity changes around the SF over time.

**Fig. S5** Using a series of STEM images (Fig.4), the strain distribution around one faulted plane could be obtained. The stacking fault induces a tensile strain in the near matrix, while the stacking faulted atomic plane receives compressive strain from the nearby matrix. Here, the tensile strain around one SF is visible and gradually reduced with irradiation. Because of the GPA method, the compressive strain in a single atomic layer could not be observed. The upper/lower panels correspond to *e_xx_* and *e_yy_*, respectively. The respective strain states have been varied under beam irradiation. On average, *e_xx_* becomes discontinued, accompanied by a slight increase of *e_yy_* around the irradiated stacking fault. Note that two additional images, at 3 min and 9 min, were added to evaluate local strain variations over time.

**Fig. S6** The quantitative analysis of planar spacing changes with time. Here, the planar spacing (the 5^th^ and 11^th^ spacings at around one stacking fault) was examined and evaluated at different times. Left: a typical HRTEM image containing one stacking fault. Right: The plot shows that the planar spacings decrease gradually over time. Note that the fit using the Gaussian function was applied.

**Fig. S7.** Corresponding strain maps to two HAADF STEM images (before and 7 minutes irradiation) in Figure 6 using GPA analysis.

**Fig. S8**	Time sequence of a series of HRTEM images recorded under a beam current density of 202 pA/cm^2^ at 200kV. No apparent damage is visible at the stacking fault sites. One stacking fault is labeled by an arrow. The FFT pattern is inserted.

**DFT calculation approach and results**

**2. Energetics**

**2.1 Formation energy (*E_f_*) of stacking fault**

**Table S1**	*Ef* in BFO

| Case | Separation (Å) | E_f_ [eV/at] N_at_ | E_tot_[eV/supercell] |
| --- | --- | --- | --- |
| 2×2×2 | 17.035 | -0.531 80 | -459.331 |
| 1×1×2 | 17.043 | -0.531 20 | -114.824 |
| BFO |  | -0.521 10 | -59.608 |

‘Separation’ means the separation of faulted planes in perfect BFO. The calculations suggest a very high SF density is expected.

**2.2 Formation energy (*E_f_*) of Vacancies**

**Table S2**	Vacancy formation energy (*Ef*) in BFO

| Case | x_vac_ | E_f.vac_ [eV] E_f_ [eV/at] | N_at_ | E_tot_[eV] |
| --- | --- | --- | --- | --- |
| 2×2×2_FR | 0.0125 | -0.426 -0.533 | 79 | -473.413 |
| 2×2×2 | 0.0125 | -0.544 -0.534 | 79 | -473.531 |
| 2×2×3 | 0.0083 | -2.569 -0.547 | 119 | -713.988 |

**2.3 Vacancy-SF interaction energies**

The interaction energies are deﬁned as:

***E_int_*** = ***E***(perf) + ***E***(SF + vac) - ***E***(SF) - ***E***(vac),

The interaction energy as a function of the plane containing the Bi vacancy is shown in **Figure 7 (**with a case of 2 × 2 × 2 (FM)).

**2.4 Vacancy-SF interaction energies under strain**

**Table S3** Bi vacancy formation energy at the faulted and nearby plane, *E_f_* [eV/at]

| Case | SF plane 1Bi-plane | | | 2Bi-plane | | SF formation in BFO | | |
| --- | --- | --- | --- | --- | --- | --- | --- | --- |
| unstrained | -0.503 -0.548 | | | -0.573 | | -0.531 | | |
| Strained | -0.535 -0.527 | | | -0.585 | | -0.480 | | |
|  | |  |  | |  | |  |  |
